# The Comparative Osmoregulatory Ability of Two Water Beetle Genera Whose Species Span the Fresh-Hypersaline Gradient in Inland Waters (Coleoptera: Dytiscidae, Hydrophilidae)

**DOI:** 10.1371/journal.pone.0124299

**Published:** 2015-04-17

**Authors:** Susana Pallarés, Paula Arribas, David T. Bilton, Andrés Millán, Josefa Velasco

**Affiliations:** 1 Department of Ecology and Hydrology, University of Murcia, Murcia, Spain; 2 Department of Life Sciences, Natural History Museum London, London, United Kingdom; 3 Department of Life Sciences, Imperial College London, London, United Kingdom; 4 Marine Biology and Ecology Research Centre, School of Marine Science and Engineering, Plymouth University, Plymouth, United Kingdom; Natural Resources Canada, CANADA

## Abstract

A better knowledge of the physiological basis of salinity tolerance is essential to understanding the ecology and evolutionary history of organisms that have colonized inland saline waters. Coleoptera are amongst the most diverse macroinvertebrates in inland waters, including saline habitats; however, the osmoregulatory strategies they employ to deal with osmotic stress remain unexplored. Survival and haemolymph osmotic concentration at different salinities were examined in adults of eight aquatic beetle species which inhabit different parts of the fresh—hypersaline gradient. Studied species belong to two unrelated genera which have invaded saline waters independently from freshwater ancestors; *Nebrioporus* (Dytiscidae) and *Enochrus* (Hydrophilidae). Their osmoregulatory strategy (osmoconformity or osmoregulation) was identified and osmotic capacity (the osmotic gradient between the animal’s haemolymph and the external medium) was compared between species pairs co-habiting similar salinities in nature. We show that osmoregulatory capacity, rather than osmoconformity, has evolved independently in these different lineages. All species hyperegulated their haemolymph osmotic concentration in diluted waters; those living in fresh or low-salinity waters were unable to hyporegulate and survive in hyperosmotic media (> 340 mosmol kg^-1^). In contrast, the species which inhabit the hypo-hypersaline habitats were effective hyporegulators, maintaining their haemolymph osmolality within narrow limits (ca. 300 mosmol kg^-1^) across a wide range of external concentrations. The hypersaline species *N*. *ceresyi* and *E*. *jesusarribasi* tolerated conductivities up to 140 and 180 mS cm^-1^, respectively, and maintained osmotic gradients over 3500 mosmol kg^-1^, comparable to those of the most effective insect osmoregulators known to date. Syntopic species of both genera showed similar osmotic capacities and in general, osmotic responses correlated well with upper salinity levels occupied by individual species in nature. Therefore, osmoregulatory capacity may mediate habitat segregation amongst congeners across the salinity gradient.

## Introduction

Of all the physiological challenges organisms face in the aquatic environment, an ability to maintain the osmotic concentration of body fluids in the face of fluctuations in the external environment is essential [1, 2]. For primarily freshwater organisms such as aquatic insects, salinity is a natural stressor that can disrupt metabolism and water balance [3], and therefore species inhabiting saline waters have developed a range of physiological mechanisms to deal with ionic fluctuations, which fall into two main strategies: osmoconformity and osmoregulation. Osmoconformers concentrate organic osmolytes intracellularly and/or extracellularly in response to increasing salinity, thus avoiding the toxicity associated with salt accumulation. In osmoregulators, the internal fluid compartment is instead strictly regulated regardless of the external osmotic fluctuation mainly by the active transport of ions via specialized organs [2, 4, 5].

Osmoregulation has been widely studied in marine organisms (e.g. [6–10]), where the vast majority of taxa are osmoconformers [5, 11]. In contrast, our knowledge of the osmotic mechanisms of organisms inhabiting inland waters is severely limited, despite the fact that information on the salinity tolerance of such taxa is essential for understanding their ecology and evolutionary history (e.g. [12–14]). Inland aquatic systems encompass a wide variety of habitats across a large salinity range, from freshwaters to hypersaline water bodies (up to six times the salinity of the sea, i.e. around 200 g L^-1^), which also differ in ionic composition [15, 16]. In addition, organisms inhabiting inland saline systems frequently experience large osmotic and ionic fluctuations, far exceeding those seen in most marine systems, as a result of freshwater input from rainfall, or evaporation during dry periods [16, 17]. As a consequence, tolerance of osmotic stress is one of the main constraints to colonization and survival in such ecosystems. Despite these challenges, specialization in saline waters has occurred in many primarily freshwater lineages [14, 18, 19], which offer an ideal comparative framework within which to study the physiological traits of related species adapted to different salinity optima.

In aquatic insects, osmotic patterns have been well documented in a range of larval Diptera (reviewed in [20]). However, in other orders such as Odonata, Hemiptera and Coleoptera, osmotic and ionic regulation patterns are much less well-known; most studies to date focusing on single, unrelated species or only on larval stages (e.g. [21–27]). The osmotic responses of aquatic organisms generally appear to correlate well with the salinity range occupied in nature. Strictly freshwater forms can hyperregulate in dilute waters, but die when the external osmotic concentration reaches or exceeds that of their haemolymph (e.g. [28, 29]), whilst in salinity-tolerant taxa two patterns have been found. Some species osmoregulate at concentrations below the isosmotic point and osmoconform at higher concentrations (e.g. [25, 29–32]). In nature these species are generally limited to external ion concentrations no greater than those found in sea water (about 1000 mOsm). In contrast, it is thought that all species that show tolerance to salinities above 1000 mOsm are efficient osmoregulators (e.g. [19, 33–38]). Osmoregulation therefore seems to be the most recurrent adaptation in aquatic insects inhabiting highly saline media [4], where the additional energetic costs required by osmoregulatory mechanisms may be compensated by the competitive release afforded by these habitats [39]. However, to date, this apparent association between osmotic capacity and species salinity ranges has never been explored from a comparative perspective within clades of closely related species whose members occupy different parts of the salinity gradient.

Coleoptera is one of the most specious insect orders in inland waters, including saline habitats, having colonized water at least 20 times from separate terrestrial ancestors [40]. Recent molecular phylogenetic analyses suggest that the aquatic Adephaga (which includes the familiar diving beetles and whirligigs) have entered the aquatic environment only once [40–42], whilst in some polyphagan families, as hydrophilids, multiple transitions from the terrestrial to the aquatic environment and back again have occurred [42–44]. As well as these shifts between media, the evolutionary history of beetles includes multiple independent transitions from freshwater to saline habitats (e.g. [14]) in which the evolution of specialized mechanisms to deal with salinity must have been crucially important. A number of 'true water beetle' genera (sensu [40], i.e. with both larvae and adults strictly aquatic) have occupied the full salinity gradient, including closely related species with contrasted habitat preferences, i.e. from strictly freshwater species to hypersaline specialists that are able to survive at salinity levels too toxic for any aquatic vertebrate [16]. Such taxa are therefore ideal models with which to explore the evolution and physiological diversity of osmotic stress mechanisms and their relation to habitat occupation. However, whilst a number of studies have explored osmoregulation in terrestrial beetles exposed to dehydration (e.g. [45–49]), information on the osmotic mechanisms of aquatic beetles in saline waters is almost entirely lacking, with only a few aquatic species having been studied, e.g. the freshwater *Dytiscus verticalis* [26, 27], larvae of freshwater *Elodes* [21] and a handful of saline water species such as *Berosus spinosus* [22] and *Hygrotus salinarius* [24]. A better understanding of the osmotic strategies of different lineages of water beetles should provide insights into the evolutionary processes of physiological adaptation to saline waters. In addition, information on species responses to osmotic stress may assist in the assessment of the potential for communities to deal with environmental change [50, 51]. In particular, studies on osmoregulation are important in the context of increasing aridity and salinization of inland waters, which may result in severe biodiversity losses [52, 53], especially in regions which already experience dry and Mediterranean climates [54].

Here we explore osmotic responses and survival to acute salinity exposure in adults of 8 water beetle species belonging to the genera *Nebrioporus* (Adephaga: Dytiscidae) and *Enochrus* (Polyphaga: Hydrophilidae). Within each lineage, we study species inhabiting the different parts of the fresh—hypersaline gradient (see Table 1). Our aims were to: 1) identify and describe species osmoregulatory strategies (i.e. osmoconformity or osmoregulation) to determine if the same mechanisms of dealing with salinity have evolved in these two genera which have independently colonized inland saline waters from freshwater ancestors and 2) compare species osmoregulatory strategies and osmotic capacities (i.e. the osmotic gradient between the animal’s internal medium and the external medium), checking for correlation with species salinity preferences in nature and for differences between co-habiting species of the two lineages.

**Table 1:** Species´ habitat and collecting sites.

	Occupied habitats			Collection sites		
Species	Conductivity range (mS cm^-1^)	Mean conductivity (mS cm^-1^)	Habitat preference*	Locality	Latitude	Longitude
*N*. *b*. *cazorlensis*	0.15–0.61	0.40	Freshwater	Río Tus, Albacete	38.3707	-2.4459
*N*. *clarkii*	0.11–9.00	1.26	Subsaline-Hyposaline	Río Corneros, Murcia	37.7173	-1.9053
*N*. *baeticus*	4.10–160.00	36.65	Mesosaline	Río Chícamo, Murcia	38.2175	-1.0511
*N*. *ceresyi*	4.50–129.00	53.68	Mesosaline-Hypersaline	Laguna Cotorrillo, Murcia	37.8251	-0.7619
*E*. *salomonis*	0.70–2.16	1.23	Subsaline	Arroyos en Laguna de Pétrola, Albacete	38.8471	-1.5589
*E*. *politus*	1.50–133.40	19.32	Hyposaline	Río Chícamo, Murcia	38.2175	-1.0511
*E*. *bicolor*	2.10–86.00	34.96	Mesosaline	Laguna Mojón Blanco, Albacete	38.8002	-1.4301
*E*. *jesusarribasi*	14.90–160.00	62.14	Hypersaline	Rambla Salada, Murcia	38.1263	-1.1182

Conductivity of the habitats of the studied species (field data from Biodiversity database of the Aquatic Ecology Research Group at the University of Murcia) and location of collecting sites.

* Ranges of conductivity of each category (mS cm^-1^): Freshwater: < 1, Subsaline: 1–10, Hyposaline: 10–30, Mesosaline: 30–60, Hypersaline: > 60 [55]

## Material and Methods

### Studied species

The Mediterranean basin hosts a wide variety of inland aquatic habitats, covering the full salinity range, from freshwater to hypersaline water bodies [16, 55, 56]. *Enochrus* (Polyphaga: Hydrophilidae) and *Nebrioporus* (Adephaga: Dytiscidae) are amongst the most common and representative genera found in water bodies across the Mediterranean region [16, 57], including species occupying different parts of the salinity gradient, with both larval and adult stages being strictly aquatic.

Within each of these genera, we selected four species with different salinity occupancy ranges in the field (see Table 1), including species that commonly are found in freshwater (*N*. *bucheti cazorlensis* (Lagar, Fresneda and Hernando, 1987)), subsaline (*E*. *salomonis* (Sahlberg, 1900)), hyposaline (*N*. *clarkii* (Wollaston 1862) and *E*. *politus* (Küster, 1849)), mesosaline (*N*. *baeticus* (Schaum 1864) and *E*. *bicolor* (Fabricius, 1792)) and hypersaline waters (*N*. *ceresyi* (Aube 1838) and *E*. *jesusarribasi* Arribas and Millán, 2013).

### Animal collection, maintenance and experimental design

Adults of each species were collected in different areas in Spain (Table 1), most of them located in public land not covered by any special legal protection. For those localities placed in protected areas, the collections were made with the corresponding permissions from the local authorities (Dirección General de Áreas Protegidas y Biodiversidad, Consejería de Agricultura y Medio Ambiente de Castilla La Mancha for Río Tus and Laguna de Pétrola and Dirección General de Medio Ambiente, Consejería de Agricultura y Agua de la Región de Murcia for Laguna Cotorrillo and Rambla Salada). None of the studied species is included in national or international lists of protected or endangered species. Specimens were maintained for one week in 7 L aquaria placed in an environmental chamber (SANYO MLR-351, Sanyo Electric Co., Ltd., Moriguchi City, Osaka, Japan) at 20°C and 12:12 L:D cycle. Each species was maintained at their optimum salinity (see mean conductivity of habitat in Table 1), using water from collection sites. Food was provided daily (chironomid larvae for *Nebrioporus* species and macrophytes for *Enochrus*).

Groups of 15–25 animals were exposed for 48 h to different salt concentrations that include the range that each of the species commonly occupies, and lower and upper extremes, as follow: 1, 3, 5, 10, 20, 40 and 50 mS cm^-1^ for *N*. *b*. *cazorlensis* and *E*. *salomonis*; 1, 5, 20, 50, 75 and 100 mS cm^-1^ for *N*. *clarkii* and *E*. *politus*; 1, 20, 50, 100, 140 and 180 mS cm^-1^ for *N*. *baeticus*, *N*. *ceresyi*, *E*. *bicolor* and *E*. *jesusarribasi* (see equivalent osmolalities in S1 Table). Pilot trials showed that haemolymph osmolality stabilized by 2 days after transfer, as has been previously shown in other studies (e.g. [32]). Waters of different conductivity were prepared by dissolving an appropriate quantity of marine salt (Ocean Fish, Prodac, Cittadella, Padua, Italy) in distilled water. Experimental aquaria (1 L capacity) were filled with 400 mL of water at the test salinity and held in the environmental chamber at constant temperature (20°C) and 12:12 L:D cycle. Food was not supplied during this period in order to avoid variation in dietary ion intake between the species. Each treatment was replicated three times for each species. Mortality was recorded after 48 h exposure and surviving animals used for haemolymph sampling.

### Measurements of haemolymph osmolality

Haemolymph samples were obtained in those treatments with ≤ 50% mortality. Specimens were rinsed in distilled water, dried on blotting paper and placed between two parafilm layers under the binocular microscope. A puncture was made in the pronotum and the resulting haemolymph droplet immediately collected with a 2 μl micro-syringe (Hamilton Company, Reno, Nevada, USA), transferred to cooled eppendorf tubes filled with type B immersion oil (Cargille Laboratories, Cedar Grove, New Jersey, USA) to avoid sample evaporation and melanisation, and stored at -80°C until osmolality measurements. Haemolymph samples from beetles of each treatment (i.e. 15–25 individuals) were pooled to produce the minimum volume of 2 μl required for osmolality measurements. The osmolality of the haemolymph was measured in a Wescor 5520 vapour pressure osmometer (Wescor Logan, Utah, USA) previously calibrated using Wescor standard solutions of 90, 290 and 1000 mOsmol. A special sample holder disc was used following manufacturer instructions for small sample volumes (2 μl). Haemolymph was previously separated from the immersion oil by centrifugation in a Sprout mini-centrifugue (Heathrow Scientific LLC, Vernon Hills, Illinois, USA). Samples of 10 μl of the experimental solutions were also measured with the standard sample disc to obtain external media osmolalities. A calibration curve was made to extrapolate the osmolalities of the two highest conductivities (140 and 180 mS cm^-1^), which exceeded the range of the osmometer. No permits or ethical approval were required for the experimental procedures.

### Data analysis

The osmotic concentration of haemolymph was plotted against external medium osmolality and compared with the isosmotic line (slope = 1) to determine if each species was an osmoconformer or osmoregulator. We also used generalized linear models (GLM) to define the relationship between haemolymph and external media osmotic concentration, assuming a gaussian error distribution and an identity link function [58]. Osmolality would scale linearly with proportional salinity in the absence of osmoregulation, and deviation from this theoretical linearity reflects the degree of osmoregulation. Therefore, linear and quadratic relationships were tested and the model that best fitted our data was selected based on lower AIC and higher deviance.

Osmotic capacity (OC) is defined as the difference between the osmotic concentration of the body fluids and that of the external medium [59]. OC represents an integrated measure of an organism’s physiological ability to compensate for the osmotic gradient that may occur between the internal and external environments [60, 61] in both hyposmotic (hyper-OC − positive values) and hyperosmotic (hypo-OC − negative values) conditions. The magnitude of this osmotic gradient across the conductivity range tested, i.e. the absolute value of osmotic capacity, was compared between *Nebrioporus* and *Enochrus* species pairs with similar salinity preferences (Table 1); i.e., *N*. *b*. *cazorlensis* − *E*. *salomonis*, *N*. *clarkii − E*. *politus*, *N*. *baeticus − E*. *bicolor* and *N*. *ceresyi − E*. *jesusarribasi*. For this, we employed two-way ANOVA with OC as the dependent variable and external medium osmolality, species and the interaction of both as factors. When the interaction of species x medium osmolality was significant, Tukey HSD post-hoc tests were used to identify the specific treatments in which OC differed amongst species. All analyses were performed with R v. 3.0.1 (R Development Core team, 2011).

Salinity tolerance limits of the species for 48 h exposure were estimated as the LC_50_ (the osmotic concentration which resulted in the death of 50% of individuals), using Trimmed Spearman–Karber analysis (USEPA TSK Programme Version 1.5).

## Results

### Pattern of osmotic regulation

**Fig 1:**
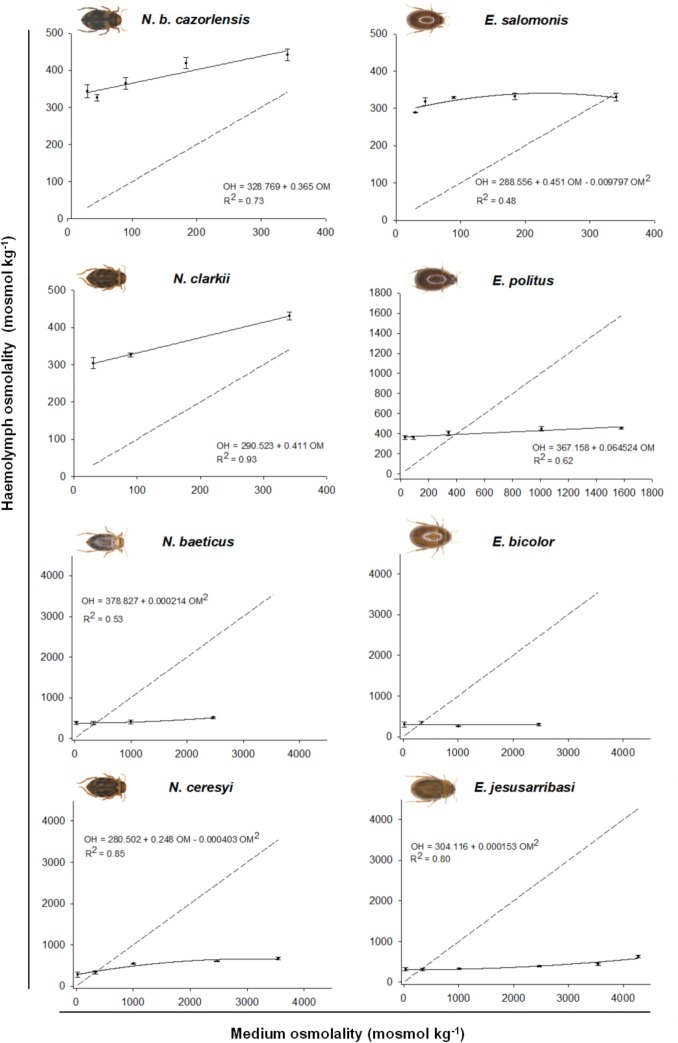
Relationship between osmotic concentration of the haemolymph and the external medium. Data are expressed as mean ± s.e. (n = 3). The isosmotic line is represented by the discontinuous line. OM: osmolality of external medium, OH: osmolality of haemolymph.

All studied species showed a capacity to hyperegulate in hyposmotic media (from 30 to 340 mosmol kg^-1^), maintaining haemolymph osmotic concentration within a range of 280–440 mosmol kg^−1^ (Fig 1)_._ The primarily freshwater *Nebrioporus b*. *cazorlensis*, *N*. *clarkii* and *E*. *salomonis* were unable to hyporegulate in media that reach or exceed their haemolymph osmotic concentration (i.e. over 340 mosmol kg^-1^), whilst the remaining saline water species (*N*. *baeticus*, *N*. *ceresyi*, *E*. *politus*, *E*. *bicolor* and *E*. *jesusarribasi*) were effective hyporegulators in hyperosmotic media. In these species, haemolymph concentration values ranged from 250 to 670 mosmol kg^−1^, across a range of external osmolalities close to lethal levels (Table 2), i.e. until 1580 mosmol kg^−1^ in *E*. *politus*, 2470 mosmol kg^−1^ in *N*. *baeticus* and *E*. *bicolor*, 3550 mosmol kg^−1^ in *N*. *ceresyi* and 4280 mosmol kg^−1^ in *E*. *jesusarribasi* (Fig 1).

**Table 2:** LC_50_ values (mosmol kg^-1^) and 95% confidence intervals estimated by the Trimmed Spearman–Karber analysis.

Species	LC50 (95%CI)
*N*. *b*. *cazorlensis*	682.68 (610.83 − 762.02)
*N*. *clarkii*	557.12 (479.47 − 644.94)
*N*. *baeticus*	2738.20 (2643.12 − 2836.65)
*N*. *ceresyi*	4190.87 (3884.61 − 4521.32)
*E*. *salomonis*	841.37*
*E*. *politus*	2249.05 (2109.71− 2400.25)
*E*. *bicolor*	3076.87 (2711.59 − 3489.81)
*E*. *jesusarribasi*	> 4280 **

* 95% confidence interval was not reliable

** mortality was lower than 50% in all tested conductivities

Positive and/or negative deviations of haemolymph osmotic concentration from the isosmotic line representing the theoretical osmolalitity of a strict osmoconformer (slope = 1), reflect the degree of hyper- and hyporegulation of the different species (Fig 1). *Nebrioporus b*. *cazorlensis* and *N*. *clarkii* had the lowest hyperegulation capacity, showing a gradual linear increase of haemolymph osmotic concentration as external medium concentration increased. In these species, the isosmotic point between haemolymph and the external medium was not reached at any of the salt concentrations tested. *Enochrus salomonis* maintained almost constant haemolymph concentration below the isosmotic point and reached this at around 300 mosmol kg^-1^, being unable to hyporegulate above this concentration. *Enochrus politus* showed a slight linear increase of haemolymph osmolality across the experimental conductivity range, but it remained both hyper and hyposmotic to the external media. Mesosaline and hypersaline species in both genera showed the strongest deviation (mainly downside) from the isosmotic line, and the relationship between haemolymph and external osmolality was non linear (see the fitted models in Fig 1) reflecting their high osmoregulatory potential. In *N*. *baeticus*, haemolymph osmolality increased nonlinearly across the conductivity gradient (Fig 1), whilst in *E*. *bicolor* it was maintained within a narrow range (255–336 mosmol kg^-1^). In the hypersaline *N*. *ceresyi* and *E*. *jesusarribasi*, haemolymph osmolality increased more markedly at the highest salinities, but in any case, hyporegulation capacity was detected until the most extreme salt concentrations tested (3550 and 4280 mosmol kg^-1^, respectively).

### Osmotic capacity

There were significant differences in osmotic capacity (OC) between the four species pairs compared, except in the case of hyposaline species (Table 3). There was also a significant species x external medium osmolality interaction, showing that species differed in their specific response patterns of OC across the range of osmotic concentrations tested.

**Table 3:** Effect of osmotality of external medium (OM), species (Sp) and their interaction on osmotic capacity (OC).

	Source	df	SS	F-value	P
Subsaline species (*N*. *b*. *cazorlensis*, *E*. *salomonis*)					
	OM	4	215826	131434	< 0.001
	Sp	1	22792	55519	< 0.001
	Sp*OM	4	7080	4312	0.011
	Residual	20	8210		
Hyposaline species (*N*. *clarkii*, *E*. *politus*)					
	OM	2	166533	106368	< 0.001
	Sp	1	2225	2842	0.118
	Sp*OM	2	5272	3368	0.069
	Residual	12	9394		
Mesosaline species (*N*. *baeticus*, *E*. *bicolor*)					
	OM	3	14582203	1290.717	< 0.001
	Sp	1	29470	7.825	0.013
	Sp*OM	3	78381	6.938	< 0.001
	Residual	16	60255		
Hypersaline species (*N*. *ceresyi*, *E*. *jesusarribasi*)					
	OM	4	38580493	3262.603	< 0.001
	Sp	1	149390	50.533	< 0.001
	Sp*OM	4	79415	6.716	0.001
	Residual	20	59125		

**Fig 2:**
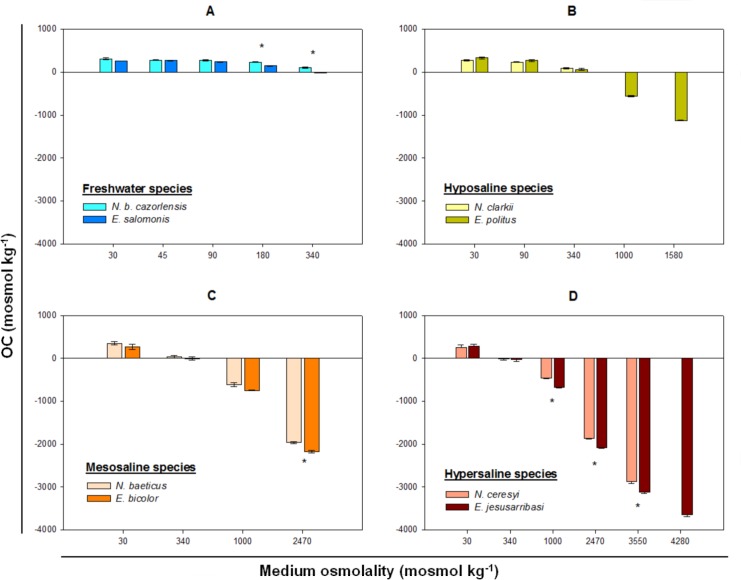
Osmotic capacities of *Nebrioporus* and *Enochrus* species pairs. Data are expressed as mean ± s.e. (n = 3). Asterisks indicate statistically significant differences between species (P ≤ 0.01) at each conductivity treatment. OC: osmotic capacity.

Species occupying fresh-subsaline waters (*N*. *b*. *cazorlensis* and *E*. *salomonis*), showed similar salinity tolerances (see LC_50_ values in Table 2) and similar mean hyper-OCs in media up to 90 mosmol kg^-1^. This was followed by a significant reduction in OC as haemolymph osmolality was closer to the isosmotic point with the external medium, at 180 and 340 mosmol kg^-1^. OC was significantly lower in *E*. *salomonis* at these osmotic concentrations (P < 0.01) (Fig 2A).

In the hyposaline species (*N*. *clarkii* and *E*. *politus*), hyper-OC showed the same decreasing tendency across an osmolality range of 30–340 mosmol kg^-1^, which was within the tolerance limits of both species (Table 2). The main differences between these species were due to the fact that *E*. *politus* was also able to osmoregulate in hyperosmotic conditions, i.e. at 1000 and 1580 mosmol kg^-1^, which were over the lethal limit of *N*. *clarkii* (Fig 2B, Table 2).

Mesosaline species displayed similar mean values of hyper-OC at 30 and 340 mosmol kg^-1^. From this concentration, hypo-OC increased with increasing osmotic stress in both species, and was significantly higher in *E*. *bicolor* than *N*. *ceresyi* at 2470 mosmol kg^-1^ (Fig 2C).

Hypersaline species showed an identical pattern of hyper-OC to mesosaline taxa in media below the isosmotic point. Above this osmolality, hypo-OC progressively increased, being significantly higher in *E*. *jesusarribasi* than in *N*. *ceresyi* across all hyperosmotic treatments (Fig 2D). In addition, this species could still osmoregulate at the highest experimental concentration (4280 mosmol kg^-1^), which was above the lethal limit for *N*. *ceresyi* (Table 2).

## Discussion

We have, for the first time, characterized the osmoregulatory strategies of adult aquatic beetles with different salinity tolerances in nature. Our study shows that species from two genera that have independently invaded saline waters are able to osmoregulate in chloride-rich waters, with no osmoconformity being observed in any of the species studied.

In media below 340 mosmol kg^−1^ (i.e. 20 mS cm^-1^), all species showed a similar pattern of hyperegulation, maintaining haemolymph concentration within a narrow range close to the typical osmolality of insect haemolymph (300 mosmol kg^−1^) [62]. Hyperegulation is a universal adaptation for life in freshwater [18], and involves the production of dilute urine to compensate for water that enters the body osmotically coupled with the replacement of lost salts by dietary intake [63] and active ion uptake [5, 20]. In most insects, Malphigian tubules and the rectum are responsible for urine formation [64, 65], and some species posses anal papillae for active ion uptake from the external environment (e.g. [21, 66–69]). In terms of the ecological implications of the osmotic patterns found here in hyposmotic media, the high survival and osmoregulatory capacity of the studied saline species in these conditions demonstrate that, at least during the adult stage, they can potentially survive in freshwaters, despite rarely being found in fresh or-low conductivity habitats in nature. This is in agreement with another recent study on saline beetle species that tolerate a wide range of salinities (including freshwater) under experimental conditions, but are restricted to waters with salinities close to their upper tolerance limits in nature [70], something also observed in saline water corixids (e.g. [23, 71]). Therefore, restriction to saline habitats may be driven by other factors such as interspecific competition and/or larval requirements (see below).

In media above the isosmotic point, only the hyposaline *E*. *politus* and the meso and hypersaline species studied were able to regulate their haemolymph osmotic concentrations. Hyporegulation capacity has previously been reported in other water beetles, as adults of the dytiscid *Hygrotus salinarius* [24], and larvae of the hydrophilid *Berosus spinosus* [22]. However, the very wide osmotic gradients that the hypersaline species here studied were able to maintain have never been demonstrated in any beetle species before. For example, the Iberian endemic species *E*. *jesusarribasi* was able to maintain its haemolymph at approximately 3500 mosmol kg^−1^ below that of the media, displaying a hyporegulation capacity comparable to those reported for some of the most effective insect osmoregulators known to date, such as the larvae of *Ephydra* brine flies [37, 38], the dolichopodid *Hydrophorus plumbeus* [72], the soldier fly *Odontomyia cincta* (Stratiomyidae) [73] or larvae and adults of some species of corixid bugs as *Trichocorixa verticalis interiores* [23] or *Sigara stagnalis* [74].

A diversity of mechanisms could be behind the extraordinary hyporegulation capacity showed by the species studied here. In general, insect adaptations to live in saline waters are designed to a) limit the entry of ions into the body and the loss of water to the external medium by osmosis, and b) actively excrete excess ions and retain water via specialized organs and tissues. The cuticle of insects represents a relatively impermeable boundary with their environment, with epicuticular lipids, and especially hydrocarbons, serving as a barrier to water loss and a waterproofing layer. However, aquatic insects seem to be in general more permeable to water than their terrestrial counterparts [5,75–77], and water loss through the cuticle has been recorded in some saline *Enochrus* species when subject to aerial desiccation (J. Velasco, unpublished data). Data on epicuticular hydrocarbons are available for a few freshwater beetle species [78, 79], but nothing is known to date regarding these in saline aquatic taxa. Despite the adaptations to minimize fluxes of water through the body wall, ion entry by drinking the external medium and feeding on food rich in salts (aquatic macrophytes and biofilms in *Enochrus* species and macroinvertebrates in *Nebrioporus*) is likely to represent an important salt input for the saline species studied here. Since access to freshwater or to food with low osmotic concentration is not available in inland saline habitats, reducing drinking rates could be a complementary behavioural adjustment to minimize ionic input [29, 80]. Such adaptations, coupled with mechanisms for active ion excretion and water conservation in specialized excretory organs likely account for the osmoregulatory capacities observed here. Insect excretory adaptations typically involve the Malpighian tubules for primary urine formation and the hindgut—the rectum in particular—as the major site of water conservation [18, 19, 81]. Osmotic and water homeostasis are often under complex hormonal control (e.g. oxytocin- and vasopressin-like peptides have been related to osmoregulatory functions in several invertebrate species [82–84]) and the specific mechanisms at morphological, biochemical and cellular levels are widely diverse between different insect groups (see 69 for an extensively review) or even between related taxa with different ecological requirements. For example, some saline-tolerant dipteran larvae possess a two-part rectum, with the posterior segment serving as a salt gland [81, 85], whilst closely related freshwater species do not show such morphological differentiation [19]. The osmotic regulation patterns reported here provide an ideal basis for further comparative studies on the specific osmoregulatory mechanisms in saline and freshwater beetles, which are so far unknown for this group of aquatic insects.

We found that species in both genera show parallel osmotic strategies in relation to the salinity ranges they occupy in nature, i.e. the species living in fresh-subsaline waters possess hyperregulation but not hyporegulation capacity, whilst species found in more highly saline waters are euryhaline osmoregulators. Osmotic capacities were similar between species of the two lineages occupying habitat with similar salinity, differing only significantly at the most elevated osmotic stress levels. Likewise, individual species were able to osmoregulate within a specific range of osmotic concentrations, which correlate with the upper salinity levels they commonly occupy in nature. This is clear, for example, in *N*. *clarkii* and *E*. *politus*. Although both species can be found in hyposaline waters, *N*. *clarkii* occupies a narrower range of salinities (Table 1) and accordingly its osmotic response and LC_50_ were similar to that of the freshwater-subsaline species, whilst *E*. *politus*, which lives within a broader salinity range, showed a similar hyper- and hyporegulation pattern to the mesosaline species *E*. *bicolor* and *N*. *baeticus*. These results together sustain the idea that within each genus, the differing osmotic capacities of the species may mediate their differential tolerances to salinity and consequently their habitat segregation across the salinity gradient [56, 70, 86].

On the other hand, despite the general concordance between field salinity and osmotic response ranges observed, our experimental data show that the saline-tolerant species studied could osmoregulate and survive at salinities that exceed both the upper and lower limits they commonly occupy in nature. The balance between the metabolic costs of osmoregulation and interspecific competition may play an important role in constraining habitat occupancy in saline waters. The osmotic stress posed by inland saline environments limits the number of species that are able to colonize them, resulting in a significant reduction in interspecific interactions, such as competition or predation in such habitats [39, 87]. The high energy demands required for homeostatic adjustment in the face of osmotic stress may result in trade-offs with other biological traits, resulting in a negative correlation between tolerance to salt and competitive ability [39, 88]. This may at least partly explain the absence of euryhaline hyper and hyporegulator species (e.g. *N*. *baeticus*, *N*. *ceresyi*, *E*. *bicolor* and *E*. *jesusarribasi*) in physiologically suitable habitats with higher species richness, such as freshwaters. In addition, however, our experiments may overestimate the true osmotic capacity of these species at salinities exceeding their natural ranges, since their regulatory mechanisms might not be maintained in the long term at these conditions [62]. Also, although larval and adult stages are truly aquatic [40] and apparently coexist during their entire life at similar salinities in nature, experimental data on salinity tolerance of larvae are lacking and it is unknown if their osmotic capacities differ to that found in adults (e.g. [24]). This could also be behind the absence of saline water species in freshwater habitats. Further studies on the osmoregulatory mechanisms and capacities of larvae would be welcome, as they may aid our understanding of the ecological and evolutionary implications of salinity tolerance in water beetles. Unfortunately such studies are hampered by a number of factors, including short larval lifespan in most species, and the taxonomic intractability of the majority of relevant larval stages.

Our understanding of the evolutionary history of colonization of saline waters by beetles is limited, but it is clear that salinity tolerance has arisen independently in a number of different aquatic lineages; for example, independent and direct transitions from freshwater to saline habitats have been reported in *Enochrus* species of the subgenus *Lumetus* [14]. Our results and previous work on osmoregulation in beetles [21, 22, 24] suggest that hyporegulation capacity has arisen in independent lineages to deal with salinity. Salt tolerance has also apparently arisen independently in larvae of many genera of mosquitoes [4, 20, 89], but in this case instead a diversity of osmotic strategies has evolved: from strictly freshwater hyperegulators, to osmoconformers and true euryhaline osmoregulators [72]. To date, the osmoconformist strategy seems to be absent amongst aquatic beetles, and generally in those lineages of insects that have successfully colonized highly saline waters [19]. Therefore, despite the fact that osmoconformity is less energetically costly [5, 90] and the most common osmotic strategy amongst marine invertebrates, osmoregulation appears as the most effective and successful adaptation to osmotic stress in insects in inland waters.

In a recent study on the evolution of salinity tolerance in *Enochrus*, [14] found evidence of multiple direct transitions to saline waters, apparently associated with periods of global aridification, as well as strong concordance between the position of species on habitat salinity and aridity gradients. The authors therefore hypothesised that the mechanisms behind salinity and desiccation tolerance might have co-evolved in this lineage. Our discovery of a generalised osmoregulation mechanism in saline water beetles is consistent with the idea of correlated evolution of such tolerances, since the physiological basis of osmoregulation has multiple commonalities with mechanisms underlying desiccation resistance [91–94]. In fact, examples of the correlation between good osmoregulatory ability and tolerance to arid conditions are abundant amongst a variety of terrestrial xeric beetles, e.g. desert tenebrionids [46, 47, 95, 96] or the meloid *Cysteodemus armatus* [97]. In the case of aquatic Coleoptera, the development of drought tolerance in lineages subjected to strong seasonal aridity may, therefore, have provided the genetic and physiological basis behind hyporegulation capacity, making colonisation and diversification in saline waters possible.

In conclusion, our findings suggest that osmoregulation could be a generalized strategy to deal with osmotic stress among adult aquatic beetles, and reveal that the evolution of enhanced hyporegulation capacities might have played a key role in the colonization of saline waters by some lineages.

## Supporting Information

S1 Table Equivalent osmolalities of the experimental conductivities. (DOC)
