# Supplementary material for: The Comparative Osmoregulatory Ability of Two Water Beetle Genera Whose Species Span the Fresh-Hypersaline Gradient in Inland Waters (Coleoptera: Dytiscidae, Hydrophilidae)
Source: PLoS One. 2015 Apr 17;10(4):e0124299. doi: 10.1371/journal.pone.0124299 (PMC4401727; doi:10.1371/journal.pone.0124299)
Supplement: S1 Table — (DOC) [file pone.0124299.s001.doc]

**Table S1. Equivalent osmolalities of the experimental conductivities.**

| Conductivity | Osmolality |
| --- | --- |
| (mS cm^-1^) | (mosmol kg^-1^) |
| 1 | 30 |
| 3 | 45 |
| 5 | 90 |
| 10 | 180 |
| 20 | 340 |
| 40 | 780 |
| 50 | 1000 |
| 75 | 1580 |
| 100 | 2470 |
| 140 | 3550 |
| 180 | 4280 |
